# Beta Rebound as an Index of Temporal Integration of Somatosensory and Motor Signals

**DOI:** 10.3389/fnsys.2020.00063

**Published:** 2020-09-02

**Authors:** Pasquale Cardellicchio, Pauline M. Hilt, Elisa Dolfini, Luciano Fadiga, Alessandro D’Ausilio

**Affiliations:** ^1^IIT@UniFe Center for Translational Neurophysiology of Speech and Communication, Italian Institute of Technology, Ferrara, Italy; ^2^Department of Biomedical and Specialized Surgical Sciences, Division of Human Physiology, University of Ferrara, Ferrara, Italy

**Keywords:** beta rebound, temporal integration, somatosensory area, motor area, median nerve stimulation (MNS), transcranial magnetic stimulation (TMS)

## Abstract

Modulation of cortical beta rhythm (15–30 Hz) is present during preparation for and execution of voluntary movements as well as during somatosensory stimulation. A rebound in beta synchronization is observed after the end of voluntary movements as well as after somatosensory stimulation and is believed to describe the return to baseline of sensorimotor networks. However, the contribution of efferent and afferent signals to the beta rebound remains poorly understood. Here, we applied electrical median nerve stimulation (MNS) to the right side followed by transcranial magnetic stimulation (TMS) on the left primary motor cortex after either 15 or 25 ms. Because the afferent volley reaches the somatosensory cortex after about 20 ms, TMS on the motor cortex was either anticipating or following the cortical arrival of the peripheral stimulus. We show modulations in different beta sub-bands and in both hemispheres, following a pattern of greater resynchronization when motor signals are paired with a peripheral one. The beta rebound in the left hemisphere (stimulated) is modulated in its lower frequency range when TMS precedes the cortical arrival of the afferent volley. In the right hemisphere (unstimulated), instead, the increase is limited to higher beta frequencies when TMS is delivered after the arrival of the afferent signal. In general, we demonstrate that the temporal integration of afferent and efferent signals plays a key role in the genesis of the beta rebound and that these signals may be carried in parallel by different beta sub-bands.

## Introduction

Somatosensory and motor areas act in concert to organize and control movements. These two cortical regions are highly interconnected (Nieuwenhuys et al., 2007; Catani et al., 2012), forming an integrated functional sensorimotor network (Lemon, 2008). During voluntary movement, the somatosensory system not only passively receives signals from the external world but also actively processes them *via* interactions with the motor system (Umeda et al., 2019). The neurofunctional integration of somatosensory and motor signals may be derived from the brain electromagnetic oscillatory dynamics recorded from the scalp. In fact, modulation of beta (15–30 Hz) and Rolandic alpha (8–12 Hz) rhythms (Salmelin and Hari, 1994b; Jensen et al., 2005; Neuper et al., 2005) have been linked to the preparation and execution of voluntary movements (Salmelin et al., 1995a; Pfurtscheller et al., 1996; Leocani et al., 1997; Cassim et al., 2001; Parkes et al., 2006) as well as imagined movements (Pfurtscheller et al., 2005).

Two types of event-related spectral perturbations (ERSPs) are generally described during the execution of movements: a power reduction in both the beta and alpha frequency bands (event-related desynchronization or ERD) and a consecutive increase of beta relative to baseline (event-related synchronization or ERS). In the beta range, the ERD is observed immediately before movement onset and is sustained throughout the movement (movement related beta decrease or MRBD; Jasper and Penfield, 1949; Salmelin and Hari, 1994a; Pfurtscheller et al., 2003; Jurkiewicz et al., 2006). Clear beta ERS follows movement cessation and exhibits a period of high amplitude, which can last for several seconds (post-movement beta rebound or PMBR; Jurkiewicz et al., 2006; Neuper et al., 2006). Although the PMBR is linked to movement execution, a rebound in beta power can also be induced in the absence of voluntary movement, such as in the case of transcranial magnetic stimulation (TMS) of the primary motor cortex (Chen et al., 1998; Aono et al., 2013; Takemi et al., 2013), passive movements (Cassim et al., 2001), or following somatosensory stimulations (Neuper and Pfurtscheller, 2001; Cheyne et al., 2003; Gaetz and Cheyne, 2006). In fact, the electrical peripheral stimulation of afferent pathways (i.e., median nerve stimulation or MNS; Salmelin and Hari, 1994a; Salenius et al., 1997) after an initial period of beta ERD is followed by a clear rebound in the same range.

These observations suggest that somatosensory reafference may play a critical role in the generation of a beta rebound. For this reason, it has been proposed that the beta rebound could represent the return-to-baseline stage following sensorimotor engagement (Müller et al., 2003). However, the relative contribution of efferent and afferent signals to its genesis and modulation remains poorly understood (Sherman et al., 2016).

**Figure 1:**
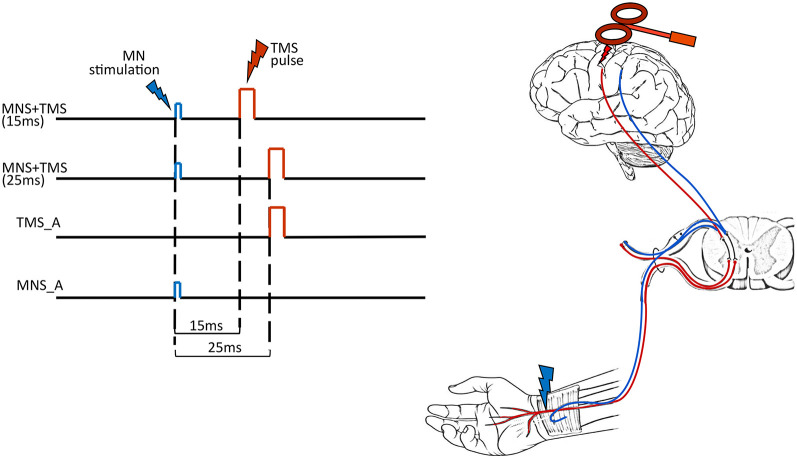
Experimental conditions. In the left panel, each row represents the timeline of each experimental condition: MNS + TMS (15 ms), MNS + TMS (25 ms), TMS alone (TMS_A) and MNS alone (MNS_A). The right panel illustrates the two types of stimulation used in the study [median nerve stimulation (MNS) in blue and transcranial magnetic stimulation (TMS) in red].

To shed some light on this issue, we combined TMS on the primary motor area (hand representation) and electrical MNS at the wrist. In the two main experimental conditions, the MNS was followed by TMS with either 15-ms (TMS15) or 25-ms (TMS25) delays. Two other control conditions are MNS alone (MNS_A) and TMS alone (TMS_A). The dependent variable was the beta rebound of the electroencephalographic (EEG) recording (Figure 1). The two main conditions (TMS15 and TMS25) tested the beta rebound modulation depending on the timing of the somatosensory afferent volley and the motor cortex stimulation. Considering that the first afferent volley requires about 20 ms to reach its cortical targets (Cohen and Starr, 1987; Allison et al., 1989), the TMS stimulation anticipated or followed the cortical arrival of the peripheral stimulus. If the peripheral stimulation arrives few milliseconds before the TMS pulse (i.e., TMS25 condition), the corresponding corticospinal excitability is reduced. This phenomenon is called short-latency afferent inhibition (SAI; Clouston et al., 1995; Manganotti et al., 1997; Fischer and Orth, 2011; Turco et al., 2018). If the peripheral stimulation arrives few milliseconds after the TMS pulse (i.e., TMS15 condition), no modulation is expected on corticospinal excitability. Still, EEG activity and the beta rebound, in particular, could differentiate if the motor cortex is stimulated after preconditioning somatosensory areas or if the afferent volley reaches its cortical targets after the preconditioning of the motor cortex. Therefore, these manipulations allow us to investigate if the relative arrival timing of afferent and efferent signals has an impact on the genesis of beta power modulation.

## Methods

### Subjects

Ten subjects (four males, six females; mean age 24.3; range 21–27) participated in the experiment. All subjects were right-handed as assessed by the Edinburgh Handedness Inventory (Oldfield, 1971) and gave informed consent to participate to the experiment. No participant had contraindication to the use of TMS (Rossi et al., 2009). The research was approved by the ethical committee *Comitato Etico Unico della Provincia di Ferrara* (approval No. 170592) and was conducted in accordance with the ethical standards of the latest updated version of the 1964 Declaration of Helsinki.

### Procedure

Subjects sat in an adjustable and comfortable armchair with a headrest ensuring a stable head position. They were instructed to keep their eyes open and to look at a fixation point located on a screen in front of them while remaining relaxed for the entire duration of the experiment. Subjects were presented with four different experimental conditions. In the first two conditions, MNS preceded TMS. These stimulations were paired and delivered at two different interstimulus intervals (ISIs): 15 ms (TMS15) or 25 ms (TMS25). The other two conditions were used as controls: TMS_A or MNS_A (Figure 1). The experiment was conducted in blocks, each one lasting ≅15 min. Each block consisted of 100 trials separated by a 5-min pause. The beta rebound following TMS or MNS is a large and easily detectable phenomenon that is present in all subjects. Here, the relatively low number of participants is counterbalanced by the large number of trials per condition to make the estimation of this effect robust within subjects. The order of blocks was pseudo-randomized across participants. The duration of the experiment was ≅75 min.

### Median Nerve Stimulation

Subjects’ right median nerves were stimulated by means of a constant-current stimulator (DS7AH, Digitimer, Hertfordshire, UK) delivering monophasic square waves of 100 μs to the volar aspect of the wrist (Figure 1) in agreement with the standard peripheral nerve stimulation montage. Cathode and anode silver chloride surface electrodes were placed with one near the other over the pathway of the median nerve in the forearm, the cathode being positioned more proximally. Stimulation intensity was adjusted for each subject to evoke a visible twitch of the thenar muscles as reported in previous studies (Salenius et al., 1997; Schnitzler et al., 1997). The stimulation intensities ranged from 5 to 12 mA. As reported by all participants, the electrical stimulation was not painful, and thus, we can infer that Aα and Aβ fibers were selectively recruited.

### Transcranial Magnetic Stimulation

A 70-mm figure-eight coil connected to a Magstim monophasic stimulator (Magstim Co., Whitland, Dyfed, UK) was placed over the left primary motor cortex with the handle pointing backward at 45° from the midline. The optimum scalp position (OSP) was found by moving the coil in 0.5-cm steps around the left primary motor cortex hand area in order to produce maximum-amplitude motor evoked potentials (MEPs) in the right *opponens pollicis* (OP) at the lowest possible intensity. The OSP was marked on a cap, and coil position was fixed by a mechanical support (Fisso Swiss Made) and continuously monitored by the experimenter. The resting motor threshold (rMT) was assessed by using standard protocols (5 out of 10 MEPs exceeding 50 μV, peak-to-peak amplitude; Rossini et al., 1994). The intensity of the TMS was then set at 120% of the rMT, and TMS stimuli were delivered every 8 ± 2 s. Mean rMT was 45% (*SD* = 6) of the maximal stimulator output.

### EMG Recordings

MEPs were recorded with a wireless EMG system (ZeroWire EMG, Aurion, Italy) from the right OP by using a standard tendon-belly montage with Ag/AgCl electrodes. EMG traces were band-pass filtered (50–1,000 Hz), digitized (2 kHz), acquired by a CED Power1401 board (Cambridge Electronic Design, Cambridge, UK), visualized, and stored on a PC for off-line analysis by means of Signal 3.09 software (Cambridge Electronic Design, Cambridge, UK).

### EEG Recording

EEG was recorded using a wireless EEG sensor cap (Enobio 8, Neuroelectrics, Barcelona, Spain) with eight EEG channels mounted according to the International 10-20 system. During acquisition, the ground and reference channel was positioned at the ear clip. EEG data were sampled at a frequency of 500 Hz (24 bit) and were acquired in continuous mode using dedicated software (NIC, Neuroelectrics, Barcelona, Spain) and exported for further analysis in the EEGLAB toolbox (Makeig et al., 1996; Delorme and Makeig, 2004). EEG data acquisition was synchronized to the internal clock of the CED Power1401 triggering the MNS and/or TMS. Electrode locations were F3, F4, C3, C4, P3, P4, Pz, and Cz.

## Data Analysis

### EMG Analysis

EMG data were analyzed for the three conditions eliciting MEPs (TMS_A and the two MNS + TMS conditions, TMS15 and TMS25). We discarded from the analysis all trials (34 trials in total) with no visible MEP (below 50 μV; mean 1%, *SD* = 1.7) or with an outlier (2 *SD*) pre-TMS EMG activity (mean 0.8%, *SD* = 1.8). MEP amplitude values were measured as peak-to-peak amplitude (in mV). The Shapiro–Wilk test was applied to test the normality of the data. Given the nonnormal distribution, we performed nonparametric statistics. To evaluate whether MEPs differed between the three protocols, we ran a two-tailed group-level permutation test (Blair and Karniski, 1993; Manly, 1997; Groppe et al., 2011). This test consists of randomly assigning, for each subject, the labels corresponding to the conditions to calculate the (group-level) difference between them. This procedure was repeated 5,000 times, generating a distribution of the differences under the null hypothesis that the probability distributions for the data belonging to each pair of conditions are mutually exchangeable. The *p*-value of the statistical test is yielded by the proportion of random permutations that results in a difference that is larger than the one observed in the original data. *P*-values were then corrected for multiple comparisons by false discovery rate (FDR; Benjamini and Hochberg, 1995). Analyses were run by using MATLAB R2013a (The MathWorks Inc., 2015).

### EEG Analysis

The EEG data was analyzed for all four conditions. The electrical artifacts associated with the TMS pulses consisted of transient high-voltage peaks. These artifacts typically lasted 3–8 ms as has also been reported in previous studies (e.g., Veniero et al., 2009; Thut et al., 2011). We removed and replaced these periods by a linear interpolation for a conservative 38-ms window around each TMS pulse (8 ms before and 30 ms after TMS onset). The data was filtered using a 1–45 Hz band-pass filter using the EEGLAB toolbox (Bell and Sejnowski, 1995; Delorme and Makeig, 2004) in MATLAB (MATLAB R2013a, The MathWorks Inc., 2015). EEG trials were segmented into 3-s epochs (−1, + 2 s from MNS stimulation or TMS stimulation in the TMS_A condition) and baseline corrected using a prestimulus baseline period (from −500 to −300 ms before the first stimulation: TMS or MNS, depending on the condition). We then removed artifact trials by using the automated artifact rejection method implemented in EEGLAB toolbox. Trials with amplitudes exceeding ± 150 μV on any of the channels were excluded from further analysis. The intra-channel kurtosis level of each epoch was computed to reject the epochs highly damaged by the noise. Those epochs containing values exceeding the average of the probability distribution of values across the data segments by 5 *SD* were rejected. The EEG data were then rereferenced to a common average reference.

Time–frequency analysis of the data was conducted using wavelet-based analysis estimating 101 linearly spaced frequencies from 5.0 to 30.0 Hz. The lowest frequency was set at 5 Hz with three cycles. At the maximum frequency (30 Hz), the cycles were nine. Inferential statistics have been conducted on C3 and C4 electrodes, which have already shown to be of interest when investigating the beta rebound (Pfurtscheller et al., 1996, 1997). We first evaluated the beta rebound as a whole. To analyze the change in beta (15–30 Hz) reactivity across conditions, we averaged the time–frequency energies across the frequency band of interest for the temporal epoch between 500 and 1,000 ms (Pfurtscheller and Berghold, 1989; Salmelin and Hari, 1994b; Stancák and Pfurtscheller, 1995; Jurkiewicz et al., 2006). We then extracted the latency and frequency of the peak beta rebound and then submitted these values to nonparametric paired-samples permutation *t-*tests. Finally, to distinguish the different contribution of low (15–20 Hz), middle (20–25 Hz), and high (25–30 Hz) beta bands, we examined the power spectra in these three bands of interest for the same epoch. Because the time–frequency energy values are likely nonnormally distributed, the differences in beta-band power, frequency, and latency between conditions were compared using FDR-corrected nonparametric paired samples permutation *t*-tests (Blair and Karniski, 1993; Manly, 1997; Groppe et al., 2011) using the same method described for the EMG data.

## Results

### EMG Results

**Figure 2:**
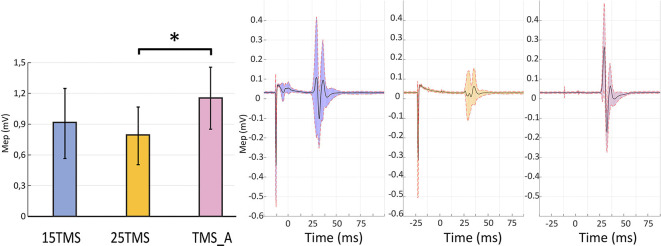
Short-latency afferent inhibition. The left panel presents motor evoked potential (MEP) amplitudes recorded in the three conditions containing TMS stimulations ±SEM: MNS + TMS (15 ms; blue), MNS + TMS (25 ms; yellow), and TMS_A (violet). An asterisk (*) on the top of two bars highlights a significant difference (permutation test; *p* < 0.05). The right panel shows the average MEP and standard deviation of the opponens pollicis (OP) muscular activity in a representative subject in each of the three conditions containing TMS.

The permutation test revealed that MEP amplitudes in the TMS_A condition (mean: 1.15 ± 0.30 mV) were significantly larger compared to those obtained when TMS was delivered 25 ms after MNS (0.79 ± 0.28; *p* = 0.01). MEP amplitudes when TMS was delivered 15 ms after MNS instead showed no significant difference with respect to TMS_A (0.91 ± 0.34 mV; *p* = 0.3; Figure 2). This result confirms the presence of a short-latency afferent inhibition of corticospinal excitability only with the 25-ms delay (Tokimura et al., 2000; Ferreri et al., 2012).

### EEG Results

**Figure 3:**
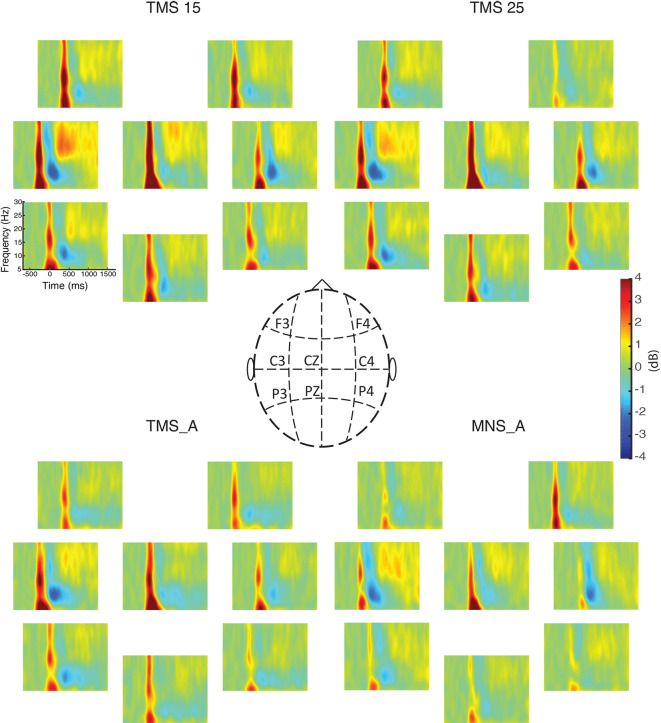
Event-related spectral perturbations (ERSPs) for all electrodes. The power spectra between 5 and 30 Hz are shown for all experimental conditions (upper left: MNS + TMS15, upper right: MNS + TMS25, lower left: TMS_A, and lower right: MNS_A). Electrode positions on the scalp are illustrated in the middle panel.

**Figure 4:**
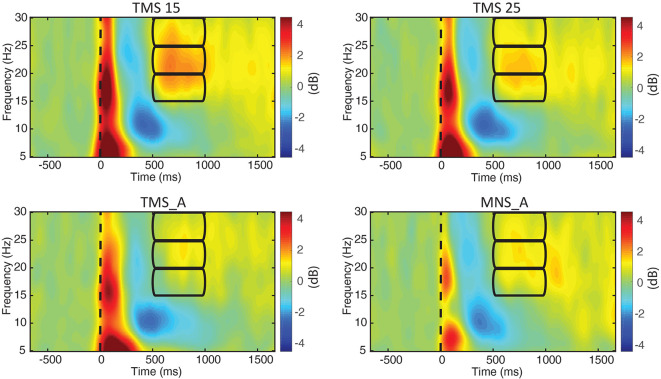
ERSPs for electrode C3. The power spectra between 5 and 30 Hz are shown for all experimental conditions (upper left: MNS + TMS15, upper right: MNS + TMS25, lower left: TMS_A, and lower right: MNS_A). Frequency bands (lower, middle, and upper beta) and time window of interest (from 500 to 1,000 ms; used thereafter for statistical comparisons) are represented by three rectangles.

We computed the grand-averaged spectrogram for all channels in each condition. The spectrogram shows an increase in beta power that occurs from 500 to 1,000 ms (Figures 3, 4). These effects peaked on central electrodes and were larger on the hemisphere contralateral for MNS. Qualitatively speaking, the time–frequency spectrograms show beta rebound modulations, depending on condition and hemisphere (Figure 3). Figure 4 shows the ERSP plot for C3 in the four conditions with a graphical representation of the spectral window of interest used in the statistical analyses.

**Figure 5:**
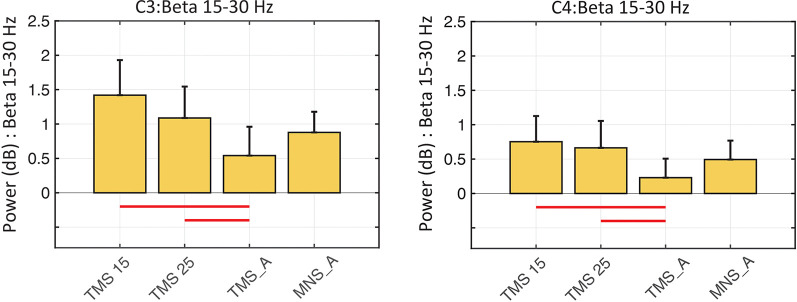
Beta rebound statistics in C3 and C4 (mean ± SEM). The histograms show the beta power rebound in a 500-ms window (from 500 to 1,000 ms) across the four experimental conditions in the C3 (left panel) and C4 (right panel) electrodes. Red horizontal lines represent significant differences between conditions (*p* < 0.05).

In both C3 and C4, the beta rebound (15–30 Hz) increased significantly in TMS15 (C3 mean: 1.41 ES ± 0.51, *p* < 0.01; C4 mean: 0.75 ES ± 0.37, *p* = 0.02) and TMS25 (C3 mean: 1.08 ES ± 0.45, *p* = 0.03; C4 mean: 0.66 ES ± 0.39, *p* < 0.01; Figure 5) compared to the TMS_A condition (C3 mean: 0.54 ± 0.41 dB; C4 mean: 0.22 ± 0.27 dB). Analyses on frequency and latency did not reveal any significant difference across conditions. Table 1 shows frequency and latency values (mean and *SD*) for both electrodes C3 and C4 in all conditions.

**Table 1:** Peak frequency and peak latency of the beta rebound, respectively, for electrodes C3 and C4.

Response	Feature	Condition	C3	C4
		TMS15	22.8 ± 1.4	22.6 ± 1.7
		TMS25	22.1 ± 1.5	24 ± 1.8
	Frequency (Hz)	TMS_A	21.2 ± 1.5	23.3 ± 1.3
Beta rebound		MNS_A	24.3 ± 1.4	22.9 ± 1.8
		TMS15	779 ± 46	793 ± 54
		TMS25	788 ± 43	710 ± 53
	Latency (ms)	TMS_A	809 ± 48	810 ± 37
		MNS_A	702 ± 36	789 ± 47

**Figure 6:**
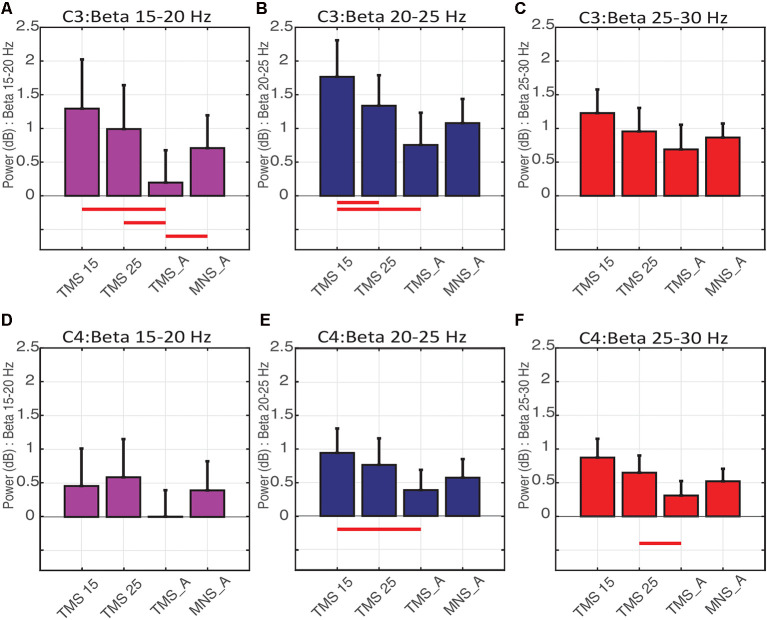
Beta rebound statistics separated into three sub-bands (mean ± SEM). The histograms show the beta rebound power in a 500-ms window (from 500 to 1,000 ms), separately for low (15–20 Hz; **A–D**), middle (20–25 Hz; **B–E**), and high (25–30 Hz; **C–F**) sub-bands. The upper panels show results for electrode C3 (**A–C**) and below for C4 **(D–F)**. Each histogram shows power values and statistical comparisons across the four experimental conditions (TMS15, TMS25, TMS_A, MNS_A). Red horizontal lines represent significant differences between conditions (*p* < 0.05).

In C3 (contralateral to MNS and on the hemisphere stimulated with TMS), the power spectra were significantly modulated by conditions on the low (15–20 Hz; Figure 6A) and middle (20–25 Hz; Figure 6B) beta bands. More specifically, low beta power in TMS15 (mean: 1.29 ± 0.73 dB, *p* = 0.02), TMS25 (mean: 0.99 ± 0.65 dB, *p* = 0.01) and MNS_A (mean: 0.70 ± 0.48 dB, *p* = 0.02) were significantly larger than in TMS_A (mean: 0.19 ± 0.47 dB; Figure 6A). No other differences were significant (TMS15 vs. TMS25: *p* = 0.17; TMS15 vs. MNS_A: *p* = 0.13; TMS25 vs. MNS_A: *p* = 0.37). In the middle beta band, power in TMS15 (1.76 ± 0.54 dB) was larger than in TMS25 (1.33 ± 0.45 dB; *p* = 0.04) and TMS_A (0.75 ± 0.47 dB; *p* < 0.01; Figure 6B). All other comparisons were not significant (TMS 15 vs. MNS_A: *p* = 0.07; TMS25 vs. TMS: *p* = 0.06; TMS25 vs. MNS_A: *p* = 0.42; TMS_A vs. MNS_A: *p* = 0.38). No differences were found in the high beta band (TMS15: 1.22 ± 0.35 dB; TMS25: 0.95 ± 0.34 dB; TMS_A: 0.68 ± 0.36 dB; MNS_A: 0.86 ± 0.20 dB; Figure 6C).

In C4 (ipsilateral to MNS), no differences were found in the low beta band (TMS15: 0.45 ± 0.55 dB; TMS25: 0.58 ± 0.56 dB; TMS_A: 0.003 ± 0.39 dB; MNS_A: 0.39 ± 0.43 dB; Figure 6D). Instead, in the middle beta band, power in TMS15 (0.94 ± 036) was larger than TMS_A (0.38 ± 0.30 dB; *p* = 0.3; Figure 6E). There were no other significant results in this band (TMS15 vs. TMS25: *p* = 0.39; TMS15 vs. MNS_A: *p* = 0.12; TMS25 vs. TMS_A: *p* = 0.13; TMS25 vs. MNS_A: *p* = 0.33; TMS_A vs. MNS_A: *p* = 0.44). In the high beta band, power in TMS25 (0.64 ± 0.25 dB) was larger than in TMS (0.30 ± 0.21 dB; *p* = 0.04; Figure 6F). No other differences were significant in this band (TMS15 vs. TMS25: *p* = 0.32; TMS15 vs. TMS_A: *p* = 0.06; TMS15 vs. MNS_A: *p* = 0.28; TMS25 vs. MNS_A: *p* = 0.13; TMS25 vs. MNS_A: *p* = 0.57; TMS_A vs. MNS_A: *p* = 0.41). Table 2 shows mean and *SD* values for both electrodes (C3 and C4) for the three beta bands recorded in all conditions.

**Table 2:** Mean power and *SD* in the three beta bands, respectively, for electrodes C3 and C4.

Power (db)	C3: 15–20 Hz	C3: 20–25 Hz	C3: 25–30 Hz	C4: 15–20 Hz	C4: 20–25 Hz	C4: 25–30 Hz
TMS15	1.29 ± 2.31	1.76 ± 1.71	1.22 ± 1.10	0.45 ± 1.75	0.94 ± 1.15	0.87 ± 0.88
TMS25	0.99 ± 2.05	1.33 ± 1.43	0.95 ± 1.10	0.58 ± 1.78	0.76 ± 1.25	0.64 ± 0.80
TMS_A	0.19 ± 1.51	0.75 ± 1.51	0.68 ± 1.15	0.00 ± 1.23	0.38 ± 0.95	0.30 ± 0.67
MNS_A	0.70 ± 1.54	1.07 ± 1.13	0.86 ± 0.65	0.39 ± 1.36	0.57 ± 0.87	0.52 ± 0.58

## Discussion

Neuronal oscillations may have an important role in regulating communication between cortical and subcortical networks (Hahn et al., 2018). Traditionally, beta oscillations have been considered as an “idling rhythm” of the motor system (Jasper and Penfield, 1949; Salmelin and Hari, 1994b; Pfurtscheller et al., 1996). The anatomo-functional origin of beta oscillations is still debated. One view suggests that these oscillations are generated in subcortical structures (basal ganglia and thalamus) and that cortical beta reflects entrainment to these inputs (Bevan et al., 2002; Courtemanche et al., 2003; Courtemanche and Lamarre, 2005). Alternatively, it has been suggested that beta oscillations are generated by the internal dynamics of cortical circuitry (Murthy and Fetz, 1992, 1996; Roelfsema et al., 1997; Brovelli et al., 2004; Jensen et al., 2005). Interestingly, the administration of benzodiazepines (enhancing GABA-A-mediated inhibition) increases beta oscillations in the human sensorimotor cortex (Jensen et al., 2005) although GABA levels in the motor cortex correlate with the beta rebound magnitude (Gaetz et al., 2011). Therefore, the circuit-level mechanisms that modulate beta power seem to be based on an “inhibitory” process, gating the transfer of information to or from SI and M1 (Sherman et al., 2016; Shin et al., 2017).

The beta rebound, in this respect, is a particularly large phenomenon appearing at the end of voluntary movements and also following peripheral stimulation with a relatively long latency (>500 ms). The nature of the beta rebound is not fully understood, but it is believed to reflect the balance of inhibition in motor networks (Salmelin et al., 1995b; Pfurtscheller et al., 1996) driven by somatosensory inputs to the motor cortex (Murray and Keller, 2011; Turco et al., 2018). Specifically, the beta rebound might reflect a “resetting” of the sensorimotor system (Engel and Fries, 2010) with the functional role of updating the nervous system with the current state of the periphery.

In the present study, we designed a series of stimulation protocols to evaluate how the beta rebound is impacted by the functional interaction and, specifically, the relative timing of motor and somatosensory signals. We used a perturb-and-measure approach to investigate the properties of the beta rhythm *via* its return to baseline (i.e., the beta rebound) in healthy participants. We applied TMS to the hand motor cortex and an electrical stimulation to the contralateral median nerve (MNS). When MNS precedes the TMS by 25 ms, the ascending signal reaches its cortical targets before the stimulation of the motor cortex. In this case, somatosensory to motor conditioning is also visible in MEP size as a reduction of corticospinal excitability (SAI; Tokimura et al., 2000; Ferreri et al., 2012). Conversely, in the 15-ms condition, the afferent signal reaches its cortical targets only after motor cortex stimulation.

Figure 3 summarizes beta power modulations produced by the different protocols in each scalp site. Modulations are mostly located in central electrodes, more prominently in the TMS-stimulated hemisphere, contralateral to the MNS stimulation. Specifically, as shown in Figure 4, the beta rebound with paired MNS + TMS is greater than that observed with TMS or MNS alone in both hemispheres. However, within the beta range, potentially independent processes (Kilavik et al., 2013) can be distinguished in upper beta (>20 Hz) and low beta rhythms (<20 Hz; Salmelin et al., 1995a; Szurhaj et al., 2003; Kilavik et al., 2012). Thus, we further analyzed the full extent of the beta band by separating it into high, middle, and low ranges.

In the left hemisphere, the upper beta (25–30 Hz) did not show any modulation to the four stimulation protocols. In the lower range (15–20 Hz), the rebound was significantly reduced in the TMS_A condition. This effect suggests that an important drive in generating the low-beta rebound is provided by the afferent signal regardless of its integration with the efferent one. In the middle range (20–25 Hz), the rebound was modulated by the delay in the paired MNS + TMS stimulations. Specifically, the rebound increased when cortical processing of the afferent signal was preceded by a stimulation to the motor cortex (TMS15). This result suggests that, in the mid-beta range, it is possible to observe state-dependency effects such that processing of the afferent volley is modulated by the preconditioning of the motor cortex. Beta rebound modulation is also induced in the right hemisphere, probably mediated by transcallosal connectivity between homolog areas. As for the motor system in which the transcallosal segment connects the motor areas (Hofer and Frahm, 2006), the activation of somatosensory areas in one hemisphere is modulated by the activity of the contralateral one (Hlushchuk and Hari, 2006; Blankenburg et al., 2008; Eickhoff et al., 2008; Kastrup et al., 2008; Klingner et al., 2011; Ragert et al., 2011; Brodie et al., 2014). In the right hemisphere, paired MNS + TMS stimulation induced greater beta rebound when compared to TMS_A. Specifically, the effect was limited to the 15-ms delay in the middle and to the 25-ms delay in the upper beta bands. Therefore, the relative timing of somatosensory and motor signals exerts opposite effects in adjacent beta bands, showing that multiple mechanisms of integration of sensory and motor signals may be at play, in parallel, in the beta band.

In this study, we show that the temporal coordination of afferent and efferent signals plays a key role in the genesis of the beta rebound. Our data suggests that motor and somatosensory areas communicate *via* spatiotemporally coordinated activities spanning multiple bands, respectively indexing the effect of efference on afferent signal processing (middle beta, contralateral to MNS) and the effect of afference on efferent signal generation (high beta, ipsilateral to MNS). Considering that our results were obtained at rest, our work shows what would be the functional relevance of having different sensorimotor integration timing across different beta sub-bands and across hemispheres during movement preparation and control.

In fact, the link between beta oscillations and movement control is quite clear. Direct manipulation of beta rhythms through the application of transcranial alternate current stimulation (tACS) abolishes the SAI (Guerra et al., 2016) and slows down movements (Pogosyan et al., 2009; Joundi et al., 2012), suggesting a causal role of sensorimotor beta oscillatory activity in motor control (Espenhahn et al., 2017). Furthermore, during sustained muscle contraction, cortical oscillations on sensorimotor regions are also phase coherent with muscle activity in the beta range (Baker and Baker, 2003). In fact, beta cortico-muscle coherence is believed to be the functional mechanism by which a bidirectional sensorimotor signal communication is established during voluntary movement (Feige et al., 2000). In support of this view, anatomical and neurophysiological evidence shows a tight integration between the neural processing of afferent and efferent signals. MNS evokes responses with almost the same latency in both areas (Lemon and van der Burg, 1979). In fact, peripheral projections reach motor and premotor neurons both directly (*via* the thalamus) and indirectly *via* S1 (Lemon and van der Burg, 1979) and, as a consequence, neurons in the motor cortex show somatosensory receptive fields (Lemon and Porter, 1976; Fetz et al., 1980). Additionally, a large portion of the descending corticospinal tract originates from somatosensory and parietal regions (Lemon, 2008) to target dorsal and intermediate spinal laminae (Morecraft et al., 2013). Finally, somatosensory areas receive information about the motor output before the arrival of sensory feedback (Umeda et al., 2019). All in all, the combination of anatomical and physiological data supports the idea that somatosensory and motor neural circuitries participate in a single functional system in the service of motor control.

However, the exact mechanism by which sensorimotor signals are integrated in time and how this is reflected in beta oscillations in different sub-bands is far from being understood. By devising a novel and relatively simple paradigm, the present study intends to provide a new tool that may be effective in clinical populations. In fact, several neurological conditions have shown altered patterns of rhythmic beta activities. For instance, multiple sclerosis patients show abnormal beta rebound (Barratt et al., 2018). Alterations in beta activity are also observed in states, such as stroke (Rossiter et al., 2014) and Parkinson’s disease (Heida et al., 2014; Little and Brown, 2014; Dubbioso et al., 2019). Considering that beta rhythms are also altered in psychiatric conditions (Liddle et al., 2016; Wessel et al., 2016; Hunt et al., 2019), our protocol opens up the possibility of testing the balance of afferent and efferent signaling as well as the efficiency of inhibitory control within sensorimotor network activity (Fry et al., 2016; Nowak et al., 2017). This result might support the development of an effective biomarker of altered neuronal communication in sensorimotor regions to improve the diagnosis of neurological and psychiatric diseases and/or to investigate the impact that drugs have on sensorimotor system functioning.

## Data Availability Statement

The datasets generated for this study are available on request to the corresponding author.

## Ethics Statement

The studies involving human participants were reviewed and approved by Comitato Etico Unico della Provincia di Ferrara. The patients/participants provided their written informed consent to participate in this study.

## Author Contributions

PC, PH, LF and AD’A had the idea and designed the experiments. PC, PH and AD’A prepared the experimental setup and collected the data. PC, ED and PH analyzed the data. All authors participated in interpretation of data and helped draft the manuscript. All authors contributed to the article and approved the submitted version.

## Conflict of Interest

The authors declare that the research was conducted in the absence of any commercial or financial relationships that could be construed as a potential conflict of interest.
